# High-Entropy (Ce_0.2_Pr_0.2_Zn_0.2_Nd_0.2_Tb_0.2_)_2_Zr_2_O_7_ Zirconate Pyrochlore: A Promising Photocatalyst for Diverse Environmental Applications

**DOI:** 10.3390/nano15211668

**Published:** 2025-11-02

**Authors:** Mariappan Anandkumar, Shanmugavel Sudarsan, Venkata Ramesh Naganaboina, Naveen Kumar Bandari, Ksenia Sergeevna Litvinyuk, Shiv Govind Singh, Evgeny Alekseevich Trofimov

**Affiliations:** 1 High-Entropy Materials Research Laboratory, South Ural State University, Chelyabinsk 454080, Russia; 2 Department of Chemistry, Saveetha Engineering College, Chennai 602105, Tamil Nadu, India; 3 Department of Electronics and Communication Engineering, Amrita Vishwa Vidyapeetham, Amaravati Campus, Amaravati 522503, Andhra Pradesh, India; 4 Department of Electrical Engineering, Indian Institute of Technology Hyderabad, Kandi 502284, Telangana, India; 5 Department of Materials Science, Physical and Chemical Properties of Materials, South Ural State University, Chelyabinsk 454080, Russia

**Keywords:** high-entropy pyrochlore oxide, Pechini synthesis, phase stability, photocatalyst, Cr(VI) reduction, dye degradation

## Abstract

Although fast-paced ongoing industrial growth, on the one hand, enhances the lifestyle of the population, on the other hand, it affects human health and the environment as a result of the discharge of pollutants. To address this, designing a novel and effective photocatalyst is necessary to mitigate increasing environmental pollutants. In the present work, we aim to synthesize a single-phase high-entropy zirconate pyrochlore oxide (Ce_0.2_Pr_0.2_Zn_0.2_Nd_0.2_Tb_0.2_)_2_Zr_2_O_7_ using a modified Pechini method. The physicochemical properties of the prepared nanoparticles were investigated using X-ray diffraction, UV-visible spectroscopy, field emission scanning electron microscopy, and X-ray photoelectron spectroscopy. The photocatalytic properties were examined using cationic dye (methylene blue), anionic dye (Congo red), and Cr(VI). Photocatalytic degradation experiments demonstrate exceptional efficiency in the removal of persistent organic pollutants. The photocatalytic results indicate that the prepared high-entropy (Ce_0.2_Pr_0.2_Zn_0.2_Nd_0.2_Tb_0.2_)_2_Zr_2_O_7_ zirconate pyrochlore oxide could effectively degrade dyes and reduce Cr(VI). Radical trapping experiments indicate that the degradation of dyes was driven by the hydroxyl radicals, superoxide radicals, and holes. Furthermore, the position of the valence band and conduction band promoted efficient photocatalytic reaction kinetics. The prepared photocatalyst remains structurally stable and can be reused three times without losing activity.

## 1. Introduction

Drinking water is of utmost importance to human survival. However, with only 3% of fresh water available to humans, fast-paced rapid industrialization further reduces the availability of fresh drinking water. This is the result of effluent being discharged straight into the water streams without being appropriately treated. To ensure the safety of human drinking water, hazardous wastewater must be treated before it is released into the environment [1]. Several dyes and heavy metals, such as hexavalent Cr(VI), are released directly into water bodies such as rivers, particularly by the dyeing, tanning, and ore processing industries [2,3,4,5]. Severe health implications have been reported when water is contaminated with dyes and heavy metals. For example, drinking water contaminated with Cr(VI) levels more than the permissible limit is likely to increase the risk of bladder, liver, kidney, and skin cancer in people [6,7]. When contamination infiltrates aquatic systems, it affects aquatic creatures, and it may indirectly affect humans when consumed [8]. Similarly, when the concentration of Congo red and methylene blue is sufficiently high, the probability of light penetration is attenuated, leading to increased biochemical oxygen demand (BOD) [9]. Apart from this, the uncontrolled release of toxic dyes may lead to severe issues like allergies, cancer, gene mutation, and lung and kidney infection [10,11].

To remediate this, it is essential to find innovative and sustainable wastewater treatment methods. Several wastewater treatment strategies, including coagulation, membrane filtration, adsorption, ion exchange, and photocatalysis, were employed to remediate contamination from dyes and heavy elements [11,12]. Among them, photocatalysis has emerged as a promising green technology for environmental remediation. Functional semiconductor materials can convert light energy into chemical energy, which facilitates the degradation of pollutants through the advanced oxidation process (AOP) [13,14]. In the AOP, reactive oxygen species (ROS), such as hydroxyl and superoxide radicals, are primarily involved in the degradation of toxic components [14,15]. Numerous photocatalytic materials are available, including metals, oxides, carbon-based materials, etc., for degrading a number of industrial dyes and heavy metals [16,17,18]. Of these, we are most interested in pyrochlore oxides, which have the general formula A_2_B_2_O_7_. In these, A-site metal cations form an eightfold coordination with oxygen, while the B-site metal cations form a sixfold coordination with oxygen anions [19]. The compositional versatility of pyrochlore-type oxides enables the easy tuning of electron/hole mobility in the lattice. As a result, pyrochlore oxides offer structural flexibility, higher oxygen mobility, and adjustable properties. These features make them a suitable candidate for catalysis, photocatalysis, solid oxide fuel cells, thermal barrier coating, and dielectric materials [20,21,22].

In general, the performance of photocatalysts depends on various factors such as particle size, bandgap, position of valence and conduction bands, recombination rate, etc. For example, TiO_2_ possesses a wide bandgap (3.2 eV), which can be excited only with UV light irradiation. In addition, during photoexcitation, the faster recombination of charge carriers explicitly slows down photocatalytic performance [23]. Therefore, modification strategies such as doping and forming heterojunctions have been employed to tune the bandgap and position of the valence and conduction bands and to delay the recombination rates. Consequently, the modified photocatalyst exhibits enhanced reaction kinetics, and a visible light source may be employed to destroy harmful contaminants. Likewise, we are particularly interested in designing a photocatalyst without doping and heterojunction formulation strategies, as a more favorable alternative to traditional reported photocatalysts, in order to simplify the synthesis process.

In recent times, the concept of high entropy has established new methods for designing a number of novel and interesting compositions for diverse functional applications. In general, high-entropy materials (HEMs) are constructed from five or more principal elements with near equimolar ratios and favor solid solution formation. High configurational entropy favors solid solution formation and hinders/lowers the possibility of the formation of secondary phases. Several classes of HEMs have been explored, including alloys, oxides, sulfides, carbides, borides, etc. [24,25,26]. In particular, high-entropy oxides designed for a pyrochlore structure offer a versatile platform for designing advanced functional materials due to their robust crystal structure and their ability to incorporate multiple cations at the A and B sites, leading to a vast compositional space and potentially novel synergistic effects.

For example, (La_0.3_Gd_0.3_Ca_0.4_)_2_(Ti_0.2_Zr_0.2_Hf_0.2_Nb_0.2_Ta_0.2_)_2_O_7_ pyrochlore oxide possesses enhanced fracture toughness and low thermal conductivity (1.45 W m^−1^ K^−1^ at 900 °C), and therefore could be suitable as a prospective thermal barrier coating material [27]. In another study, 20 new compositions of high-entropy pyrochlore were designed, while eight compositions formed a single-phase structure, and their physical properties were explored [8]. The thermal diffusivity properties of (La_0.2_Y_0.2_Gd_0.2_Nd_0.2_Sm_0.2_)Zr_2_O_7_ were investigated and found to be approximately 0.5 mm^2^/s, which could make it a potential candidate for a thermal barrier coating material [28]. Very limited literature exists on high-entropy pyrochlore oxide used for photocatalytic applications. Du et al. investigated the photocatalytic applications of (La_0.2_Nd_0.2_Sm_0.2_Gd_0.2_Y_0.2_)_2_Zr_2_O_7_ in terms of the degradation of Rhodamine B dye. Photocatalytic activity was enhanced with a decrease in particle size [29]. Recognizing that the exploration in this area has been very limited, we would like to take advantage of high-entropy pyrochlore oxide for photocatalytic applications.

Therefore, this study focuses on the synthesis and characterization of a novel high-entropy (Ce_0.2_Pr_0.2_Zn_0.2_Nd_0.2_Tb_0.2_)_2_Zr_2_O_7_ zirconate pyrochlore and investigates its potential as a multifunctional photocatalyst for environmental remediation applications, including the degradation of organic pollutants and the reduction of quantities of heavy metal ions.

## 2. Materials and Methods

### 2.1. Materials and Reagents

Cerium(III) nitrate hexahydrate (Ce(NO_3_)_3_·6H_2_O, 99.99%, Sigma Aldrich, Moscow, Russia), praseodymium(III) nitrate hexahydrate (Pr(NO_3_)_3_·6H_2_O, 99.9%, Sigma Aldrich), zinc nitrate hexahydrate (Zn(NO_3_)_2_·6H_2_O, 98%, Sigma Aldrich), neodymium(III) nitrate hexahydrate (Nd(NO_3_)_3_·6H_2_O, 99.9%, Sigma Aldrich), terbium(III) nitrate hexahydrate (Tb(NO_3_)_3_·6H_2_O, 99.99% Sigma Aldrich), Zirconium(IV) oxynitrate hydrate (ZrO(NO_3_)_2_·H_2_O, technical grade, Sigma Aldrich), Ethylene glycol (EG), and citric acid (CA). All the materials were used as received, without any further purification. Deionized water (DI) was used for the synthesis.

### 2.2. Synthesis of (Ce_0.2_Pr_0.2_Zn_0.2_Nd_0.2_Tb_0.2_)_2_Zr_2_O_7_ Pyrochlore Oxide Nanoparticles

**Scheme 1:**
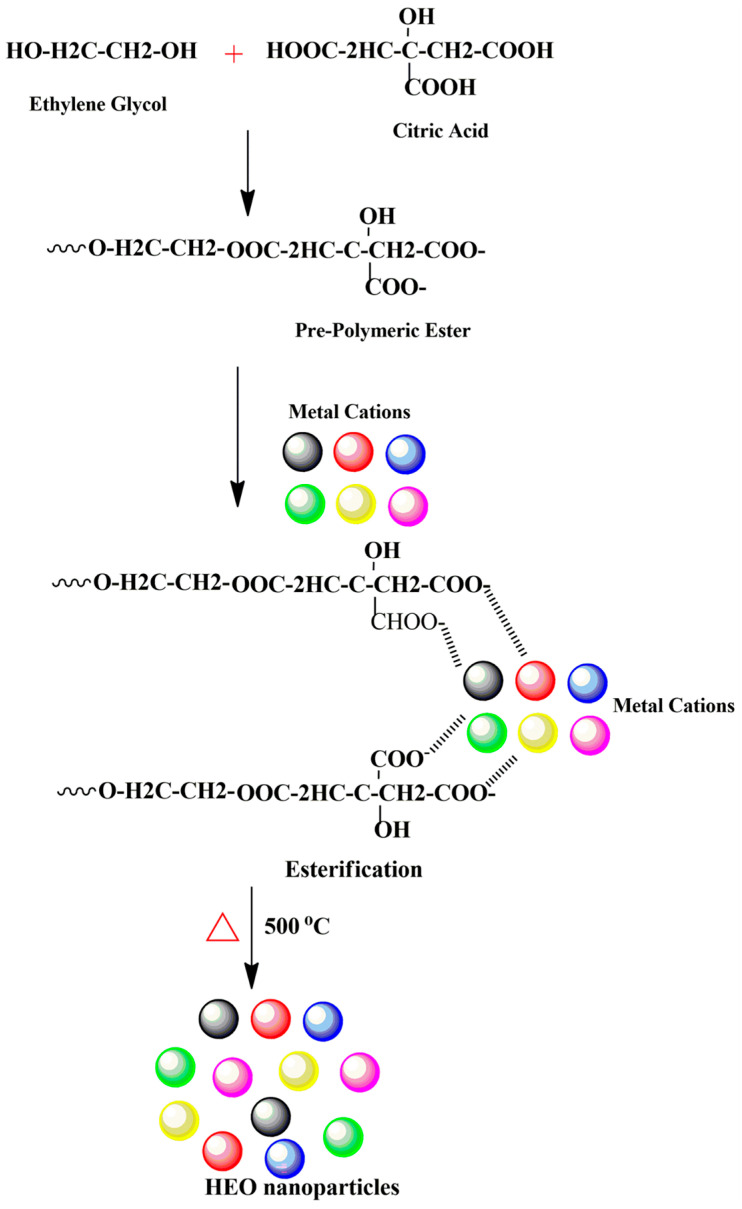
Schematic displaying the synthesis of high-entropy oxide nanoparticles prepared using the modified Pechini method.

The modified Pechini method was followed to synthesize (Ce_0.2_Pr_0.2_Zn_0.2_Nd_0.2_Tb_0.2_)_2_Zr_2_O_7_ nanoparticles [30]. Initially, 0.001 moles of individual metal salts were weighed and mixed together in a 250 mL glass beaker. Five mL of distilled water was added to the salt mixture and stirred continuously, followed by the addition of citric acid (0.576 g) and ethylene glycol (3.35 mL). The temperature of the hotplate was fixed at 110 °C to initiate the esterification reaction, and the reaction continued until a viscous gel formed. The resultant gel was dried at 300 °C in an oven for 2 h, followed by calcination at 500 °C for 4 h, and cooled naturally to room temperature to remove organics. The calcined powders were used for further characterization and photocatalytic investigations. The process of nanoparticle formation is described in Scheme 1. Overall, two chemical reactions occur: the chelation reaction between metal cations and citric acid and polyesterification with ethylene glycol. The advantage of using this synthesis technique stems from the ability to form a solid solution containing more principal elements while maintaining good elemental stoichiometry.

### 2.3. Characterization

XRD measurements were performed on the powder samples to investigate the nature of the phase formed using a powder XRD diffractometer. The powdered samples were scanned from 20° to 80° with a scan speed of 5° per minute. Williamson–Hall (W-H) analysis was performed to assess the contribution of crystallite size and the lattice strain in XRD peak broadening. FESEM images were captured using a JEOL (JEOL JSM-7001F, JEOL, Peabody, MA, USA) microscope operated at 20 kV. To investigate the electronic structure, ultraviolet photoelectron spectroscopy (UPS) was performed using a He discharge UV lamp (He I source at 21.2 eV, AXIS SUPRA, Kratos Analytical, a Shimadzu Group company, Manchetser, UK). High-resolution Raman spectral analysis was performed to investigate the structural and chemical properties of the material using a 532 nm laser connected to a WITec Alpha 300R confocal Raman microscope (WITec Alpha 300R, WITec, Ulm, Germany). X-ray photoelectron spectroscopy (XPS, K-alpha, Kratos Analytical, a Shimadzu Group company, Manchetser, UK) was performed using a monochromated Al Kα X-ray source (ℏν = 1486.6 eV), a 180° double-focusing hemispherical analyzer with a 128-channel detector, and a dual-beam flood gun.

### 2.4. Photocatalytic Investigation

For the photocatalytic investigations, we utilized a batch reaction containing dyes/Cr(VI) and a photocatalyst. Briefly, 50 mL of dye/metal ion (Congo red (50 ppm), Methylene blue (50 ppm), and Cr(VI) (50 ppm)) were added separately to a 100 mL glass beaker. Next, 25 mg of photocatalyst was added to the above solution. Then, 0.5 mL of H_2_O_2_ was added to the individual dye solutions, and 1 mL of formic acid was added to the Cr(VI) solution. The mixture was stirred in the dark for 30 min to achieve an adsorption–desorption equilibrium. Next, the solution was irradiated under a UV light source (λ = 365 nm, 100 W, Guangzhou Jiguang Lighting Co., Ltd., China) to initiate the photocatalytic reaction. Meanwhile, to investigate the photocatalytic activity of the photocatalyst, stock solutions were drawn at regular intervals (20 min) from the solution, centrifuged (5000 rpm for 10 min), and the supernatant was monitored using a UV-visible spectrophotometer (Shimadzu UV-2700, Shimadzu, Japan). The decrease in the absorbance monitored at 353 nm for Cr(VI), 499 nm for CR, and 664 nm for MB was used to estimate the degradation/reduction behavior.

## 3. Results

### 3.1. Physicochemical Properties

**Figure 1:**
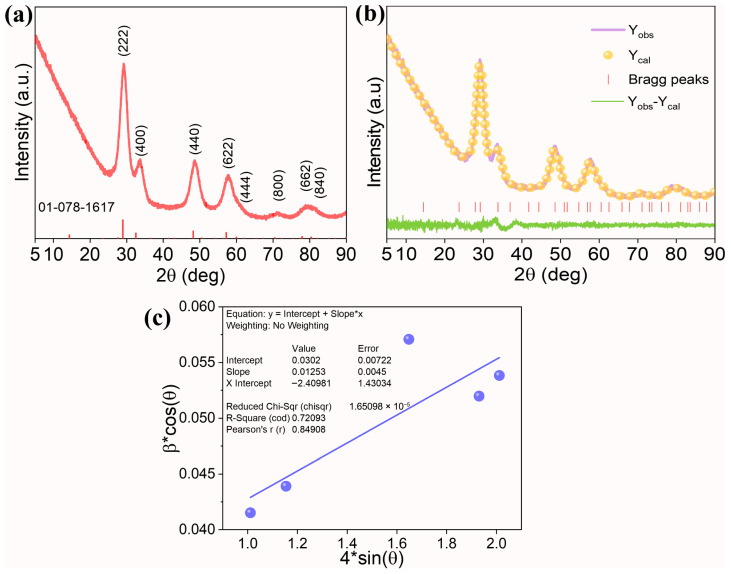
(**a**) XRD pattern of (Ce_0.2_Pr_0.2_Zn_0.2_Nd_0.2_Tb_0.2_)_2_Zr_2_O_7_ nanoparticles, (**b**) Rietveld refinement plot of the experimental XRD data, and (**c**) W-H plot to estimate the crystallite size and microstrain in the system. In the plot, “*” represent a multiple sign.

The XRD diffractogram was acquired to investigate the crystal purity and structure (Figure 1a). The strong diffraction peaks observed around 29.2°, 33.6°, 48.7°, 57.6°, 60.5°, 71.2°, 78.8°, and 81.6° were indexed to a cubic pyrochlore structure (ICDD Card No: 01-078-1617, Nd_2_Zr_2_O_7_) containing planes (222), (400), (440), (622), (444), (800), (662), and (840), respectively. In addition, no other diffraction peaks were observed, indicating good solubility of principal elements into the pyrochlore lattice, facilitating the formation of a single-phase solid solution. In general, to form a stable pyrochlore oxide, the ratio of A-site cation to B-site cation (r_A_/r_B_) must be in the range of 1.46 to 1.78 [20]. Except for Zn^2+^, all A-site cations have an oxidation state of +3 with a coordination number of 8, whereas B-site cations have a +4 oxidation state and six-fold coordination. In the current system, based on the ionic radii of, r(Ce^3+^) = 1.143 Å, r(Pr^3+^) = 1.126 Å, r(Zn^2+^) = 0.900 Å, r(Nd^3+^) = 1.109 Å, r(Tb^3+^) = 1.040 Å, and r(Zr^4+^) = 0.720 Å, a value of 1.477 was obtained, indicating a higher possibility of pyrochlore formation.

Rietveld refinement was performed on the raw XRD data using a pyrochlore structure (space group: Fd-3m) and the atomic positions of the A-site cations (0.5, 0.5, 0.5), B-site cations (0,0,0), O1 (0.35, 0.125, 0.125), and O2 (0.375, 0.375, 0.375). The output of the refinement results is displayed in Figure 1b and Table 1. The experimental data fits well with the calculated structure, underscoring the formation of single-phase high-entropy pyrochlore oxide. The lattice parameter of 10.594 Å is calculated from the Rietveld refinement result. While using the W-H plot (Figure 1c), the crystallite size and microstrain of 4.5 nm and 1.25% are obtained. Higher values of microstrain result from the contribution of different metal cations possessing different ionic radii trying to accommodate into the pyrochlore lattice. Moreover, the ionic radii of Tb and Zn are smaller than Nd, resulting in a reduced lattice parameter compared to the standard Nd_2_Zr_2_O_7_ oxide (10.676 Ả).

**Figure 2:**
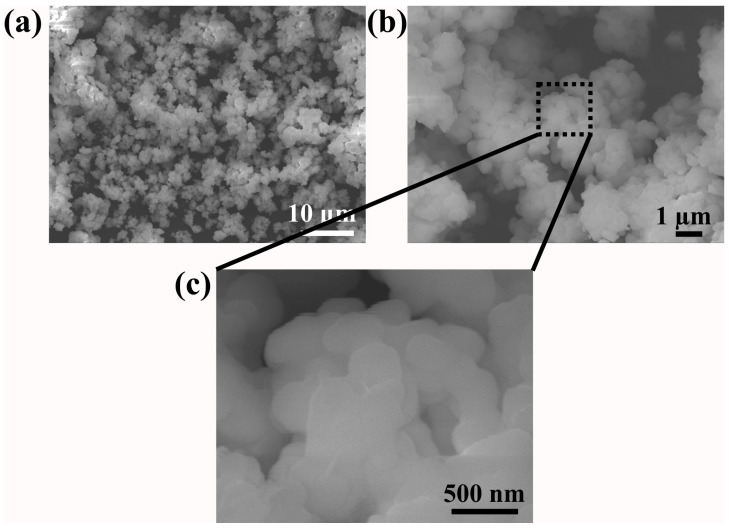
FESEM image of prepared (Ce_0.2_Pr_0.2_Zn_0.2_Nd_0.2_Tb_0.2_)_2_Zr_2_O_7_ zirconate nanoparticles: (**a**) low magnification, (**b**) high magnification, and (**c**) enlarged image.

The surface morphology of the prepared high-entropy zirconate was examined using FESEM (Figure 2). Highly agglomerated nanoparticles with an irregular and asymmetric shape are observed in the calcined samples. In general, nanoparticles possess high surface area and surface energy. During the calcination process, the available thermal energy drove the particle size to increase through a diffusion process by consuming the neighboring particles, thus lowering the surface energy [31]. This leads to particle agglomeration, increasing its size, which is observed in the present study.

**Figure 3:**
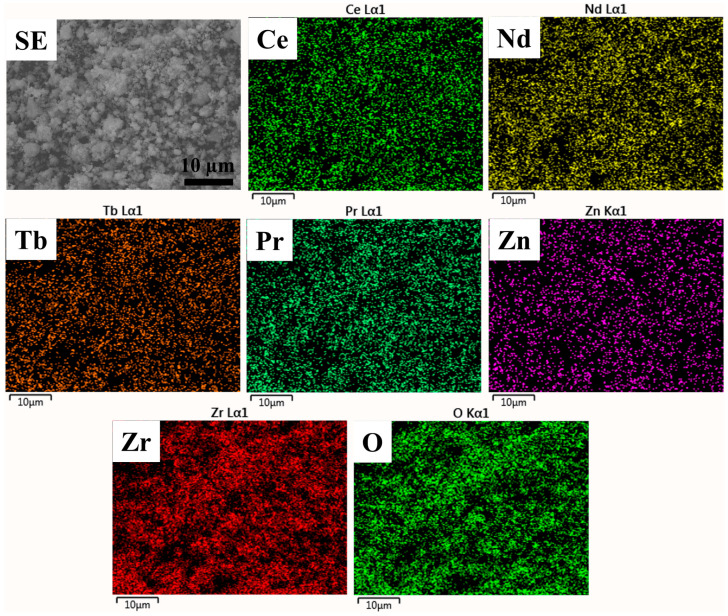
EDS elemental mapping of prepared (Ce_0.2_Pr_0.2_Zn_0.2_Nd_0.2_Tb_0.2_)_2_Zr_2_O_7_ zirconate nanoparticles. The scale bar represents 10 µm.

Since high-entropy oxide contains multiple principal elements, it is necessary to investigate the nature of elemental distribution. Here, EDS mapping was carried out to investigate the nature of the elemental distribution of the prepared high-entropy (Ce_0.2_Pr_0.2_Zn_0.2_Nd_0.2_Tb_0.2_)_2_Zr_2_O_7_ zirconate nanoparticles (Figure 3). The results indicate that the prepared nanoparticles contain an even distribution of principal elements, underscoring the formation of a solid solution. Moreover, the elemental quantification estimated from the EDS spectra (Table 2) indicates near stoichiometry of the elements designed for the pyrochlore oxide system. Overall, the Pechini method would be a viable synthesis technique to produce single-phase high-entropy oxide systems.

**Figure 4:**
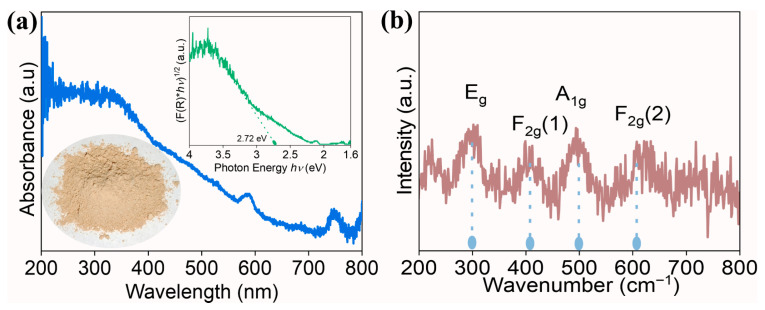
(**a**) UV-visible absorption spectra of high-entropy (Ce_0.2_Pr_0.2_Zn_0.2_Nd_0.2_Tb_0.2_)_2_Zr_2_O_7_ zirconate nanoparticles, (inset) K-M plot to estimate the bandgap and visual appearance of the prepared nanoparticles, and (**b**) Raman spectrum recorded on high-entropy (Ce_0.2_Pr_0.2_Zn_0.2_Nd_0.2_Tb_0.2_)_2_Zr_2_O_7_ zirconate nanoparticles.

Optical properties of the prepared oxide were investigated using UV-visible spectroscopy, and the plot is displayed in Figure 4. The spectrum shows a well-defined absorption band in the UV region from 400 nm, which arises due to the promotion of electrons from O^2−^ to M^3+/4+^ during photon interaction. To estimate the bandgap, Kubelka-Munk (K-M) analysis (inset of Figure 4) was performed, and the bandgap value of 2.72 eV was obtained. Compared to pristine oxides, the obtained bandgap is lower, and this can be attributed to either quantum confinement or oxygen defects. In general, high-entropy oxides contain a number of principal elements and demonstrate synergistic interactions. Moreover, in the current investigation, elements such as Ce and Pr usually coexist in +3 and +4 oxidation states. The mixed valence nature of elements facilitates the creation of oxygen vacancies through charge compensation [32]. These bandgap values will assist in estimating the conduction band energy and valence band energy later on.

Raman spectroscopy was conducted to identify the phase purity of the prepared high-entropy (Ce_0.2_Pr_0.2_Zn_0.2_Nd_0.2_Tb_0.2_)_2_Zr_2_O_7_ zirconate nanoparticles. This stage provides information regarding lattice disorder, especially with regard to oxygen anions, which are highly sensitive to the local environment. In general, according to the group theory, pyrochlore oxides possess six active Raman vibrational modes, including (A_-g_ + E_g_ + 4 F_2g_). However, only four modes Eg, F2g(1), A1g, and F2g(2) exist for typical pyrochlore systems [33,34]. In our system, we observe four Raman vibrations centered around 296 cm^−1^, 405 cm^−1^, 495 cm^−1^, and 605 cm^−1^, corresponding to Eg, F2g(1), A1g, and F2g(2), respectively. No additional Raman modes are present, demonstrating the phase purity of the prepared high-entropy (Ce_0.2_Pr_0.2_Zn_0.2_Nd_0.2_Tb_0.2_)_2_Zr_2_O_7_ zirconate nanoparticles. We observed a shift in Raman vibrations compared to the reported pyrochlore oxides [34]. This is attributed to the variation in chemical bonds existing between different metal cations and the oxygen anions.

### 3.2. Photocatalytic Reaction

**Figure 5:**
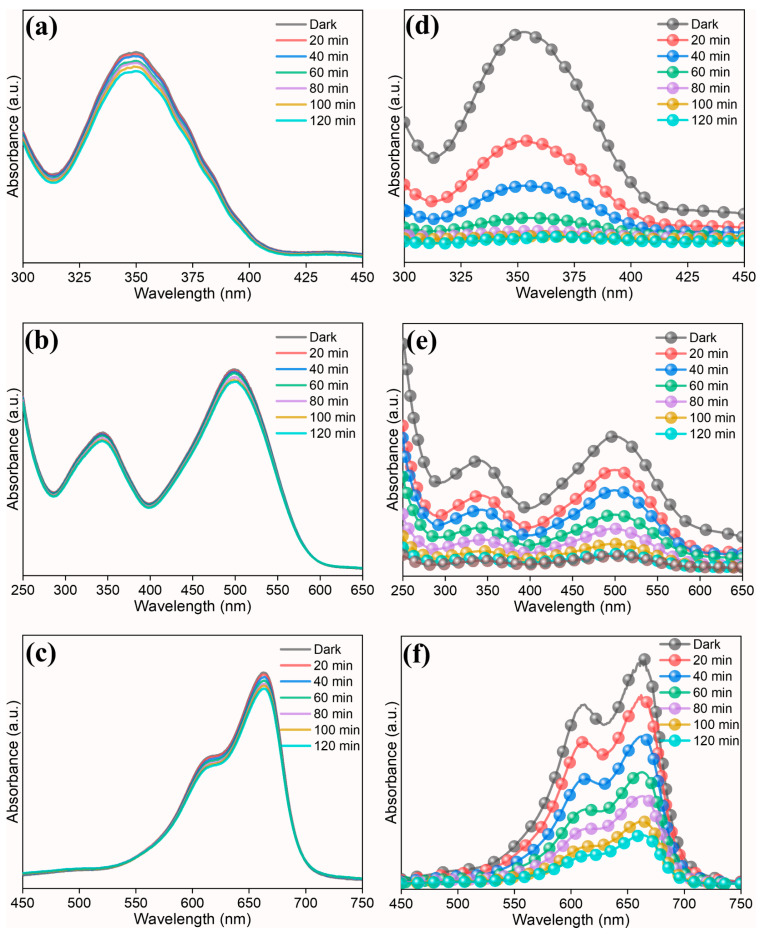
UV-visible spectra showing photocatalytic reaction. (**a**,**d**) Cr(VI), (**b**,**e**) Congo red, (**c**,**f**) methylene blue. (**left**—control experiment without photocatalyst and **right**—with photocatalyst).

To investigate the photocatalytic properties of prepared high-entropy (Ce_0.2_Pr_0.2_Zn_0.2_Nd_0.2_Tb_0.2_)_2_Zr_2_O_7_ zirconate nanoparticles, two model pollutants (anionic dye–Congo red and cationic dye—methylene blue) and a heavy metal (Cr(VI)) were chosen. Since the bandgap of the prepared nanoparticle possesses good absorbance behavior in the UV region, we have used a UV light source for the photocatalytic studies. Figure 5a–c display a control experiment in the absence of a photocatalyst. The impact of photolysis on the degradation of dyes and Cr(VI) is low; however, when a photocatalyst is added (Figure 5d–f), one can observe the degradation of dyes and reduction in Cr(VI). This proves that the prepared high-entropy oxide acts as a photocatalyst, improving the overall reaction kinetics.

**Figure 6:**
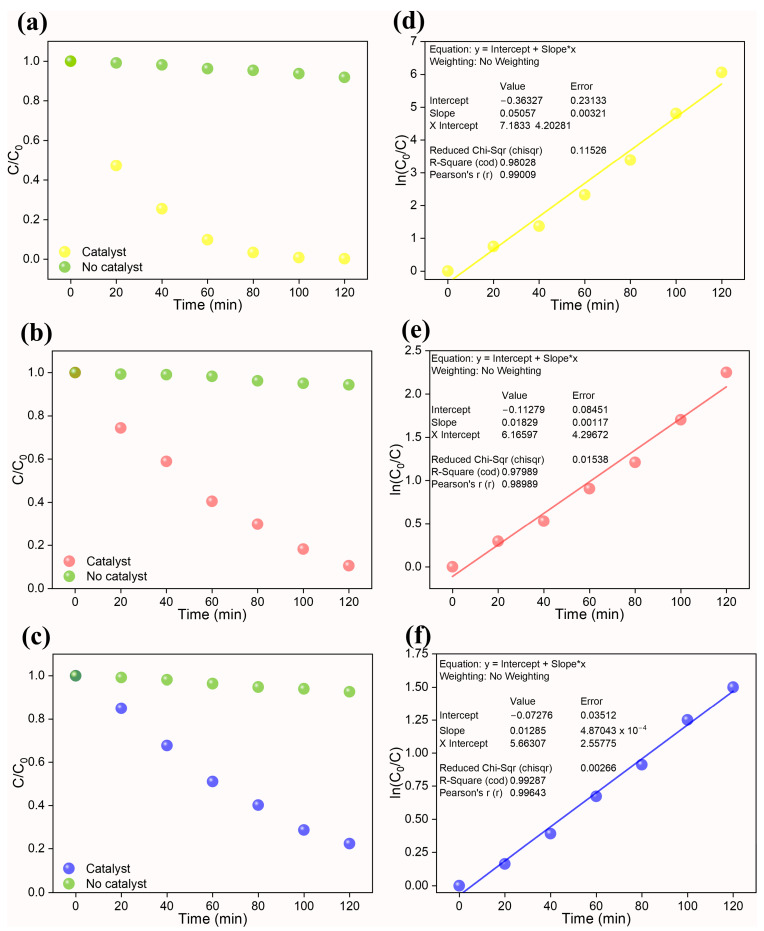
**Left**—C/C_0_ plots of (**a**) Cr(VI), (**b**) Congo red, and (**c**) methylene blue and **Right**—their corresponding pseudo-first-order kinetic plot ((**d**) Cr(VI), (**e**) Congo red, and (**f**) methylene blue). In the plot, “*” represent a multiple sign.

In the current photocatalytic investigation, the reaction kinetics were investigated using a pseudo-first-order reaction given by k*t = ln(C_0_/C). The plot C/C_0_ plot during the photocatalytic reaction is displayed in Figure 6a–c. Here, C corresponds to the concentration of dye/Cr(VI) at time t, while C_0_ corresponds to the concentration of dye/Cr(VI) at time t = 0. The rate constant (k) was estimated from the slope of the pseudo-first-order reaction kinetic fit (ln(C_0_/C)), and the results are tabulated in Table 3.

The k values of 0.0506 min^−1^, 0.0183 min^−1^, and 0.0128 min^−1^ were obtained, respectively, for, Cr(VI), CR, and MB dye. Based on the rate constant values, the prepared high-entropy pyrochlore oxide showcased better photocatalytic activity toward the degradation of dyes and reduction in Cr(VI) ions. A comparison table (Table 4) is presented to evaluate the performance of the synthesized photocatalyst with respect to reported photocatalysts. The performance of recently reported photocatalysts was lower than that of our high-entropy (Ce_0.2_Pr_0.2_Zn_0.2_Nd_0.2_Tb_0.2_)_2_Zr_2_O_7_ oxide photocatalyst. In the majority of the described studies, heterojunction photocatalysts were constructed. This emphasizes the necessity of high-entropy materials for prospective photocatalytic applications. Further research directions for the research community would be modifying the high-entropy oxides by combining them with other metal oxides to form heterojunctions to tailor the functional properties.

### 3.3. Proposed Photocatalyst Mechanism

**Figure 7:**
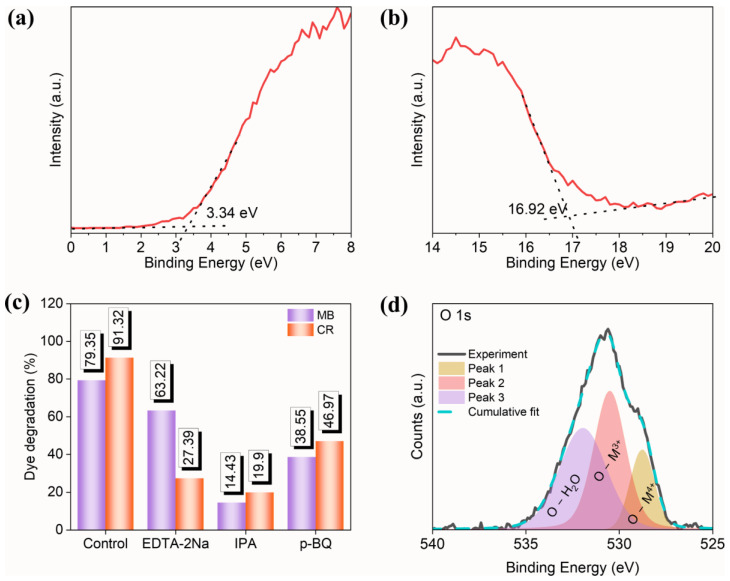
UPS spectra of (Ce_0.2_Pr_0.2_Zn_0.2_Nd_0.2_Tb_0.2_)_2_Zr_2_O_7_ zirconate nanoparticles at (**a**) low binding energy region and (**b**) high binding energy region, (**c**) radical trap experiment using various quenchers, and (**d**) XPS core spectra of O1s.

To investigate the photocatalytic reaction mechanism, it is necessary to estimate the conduction band energy and valence band energy of the prepared high-entropy (Ce_0.2_Pr_0.2_Zn_0.2_Nd_0.2_Tb_0.2_)_2_Zr_2_O_7_ zirconate pyrochlore oxide nanoparticles. The reaction kinetics of typical semiconductor photocatalysts depend on the position of the valence band (Evb) and the conduction band (Ecb) [46]. The optimal positions of the Ecb and the Evb decides the selectivity of free radical generations, which are mostly involved in the degradation of dyes, especially in the generation of reactive oxygen species. Each reactive oxygen species has a distinct redox potential, and the positions of the Ecb and the Evb are crucial and affect the photocatalytic activity. Ultraviolet photoelectron spectroscopy (UPS) analysis was carried out to identify the Evb and Ecb positions (Figure 7a,b). With the help of the bandgap (estimated from the UV-visible plot) and the UPS plot, the work function, Ecb, and Evb with reference to vacuum energy were found to be 4.3 eV, 2.35 eV, and 5.07 eV, respectively. For photocatalytic investigations, the reference must be with respect to NHE, and the conversion of (E_NHE_ = E_vacuum_ − E_NHE, absolute_), where E_NHE_ is the potential with respect to NHE, E_vacuum_ is the potential with respect to vacuum, and E_NHE, absolute_ is the absolute potential of NHE with respect to vacuum (4.44 eV) [47]. Therefore, the band positions of the Ecb and the Evb are −2.09 V and 0.63 V vs. NHE.

Radical trapping experiments were carried out to identify the selectivity of free radical species involved in the degradation of MB and CR dye. Before initiating the photocatalytic reactions, 1 mM each of isopropyl alcohol (IPA), p-benzoquinone (p-BQ), and ethylenediaminetetraacetic acid disodium salt (EDTA 2Na) were added to the reaction mixture separately. During the photocatalytic reactions, IPA, p-BQ, and EDTA 2Na act as scavengers towards hydroxyl radicals, superoxide radicals, and holes, respectively. The radical trapping results (Figure 7c) indicate that degradation is facilitated by hydroxyl radicals, superoxide radicals, and holes.

When the photocatalyst is irradiated with a light source whose energy (hʋ) is larger than the bandgap values (E_g_), electron–hole pairs are produced (Equation (1)). Next, the photogenerated holes (h^+^) stay in the valence band while the electron (e^−^) is placed in the conduction band of the photocatalyst. The lifetime of an electron–hole pair is too small, and most of the generated electron–hole pairs are recombined, hindering the reaction kinetics. To prevent the unrewarding recombination process, either an electron scavenger or a hole scavenger must be added to facilitate faster reaction kinetics [48,49]. Here, H_2_O_2_ serves as a source of hydroxyl radicals for the degradation of CR and MB dyes, which are produced by interaction with electrons accessible in the conduction band (Equation (2)). Similarly, holes interact with adsorbed water to form hydroxyl radicals (Equation (3)). Superoxide radicals are generated when adsorbed water is reduced by the available electrons in the conduction band (Equation (4)). Likewise, the contribution of oxygen vacancies cannot be neglected, as they play an important role in photocatalytic reactions. The oxygen vacancies were investigated for the synthesized high-entropy (Ce_0.2_Pr_0.2_Zn_0.2_Nd_0.2_Tb_0.2_)_2_Zr_2_O_7_ pyrochlore zirconate oxide using XPS analysis (Figure 7d). The high-resolution oxygen spectrum contains three peaks at 532.94 eV, 531.50 eV, and 529.76 eV, labeled to surface hydroxyl groups (adsorbed water), oxygen vacancies, and lattice oxygen, respectively. Accordingly, 39.84% of oxygen vacancies are available in the prepared system. Metal oxides containing aliovalent cations inherently possess anionic vacancies compensating for charge neutrality. These vacancies have a favorable influence on the bandgap, and they may act as charge-trapping centers to capture charge carriers [50,51].

**Figure 8:**
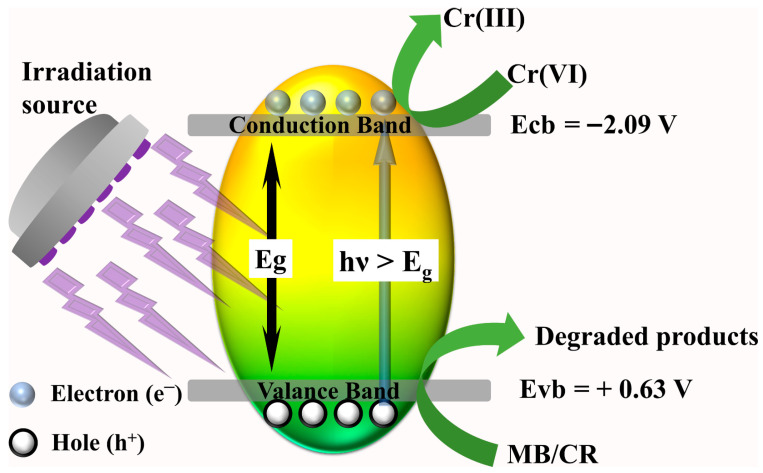
Proposed mechanism for the degradation of dyes and reduction in Cr(VI) by a high-entropy (Ce_0.2_Pr_0.2_Zn_0.2_Nd_0.2_Tb_0.2_)_2_Zr_2_O_7_ pyrochlore zirconate oxide photocatalyst.

Therefore, by effectively engineering oxygen vacancies of metal oxides, one could tailor the luring ability of holes at the valence band of the semiconductor. As a result, the lifetime charge carrier recombination is delayed, facilitating favorable reaction kinetics. Cumulatively, the produced free radicals successfully target the dye molecules and decompose them (Equation (5)). In the case of photocatalytic reduction in Cr(VI), available holes are scavenged by the added formic acid, forming CO_2_ and H^+^ (Equation (6)). This process creates a surplus of available electrons, and the process of Cr(VI) reduction to less toxic Cr(III) occurs as shown in Equation (7). Overall, the favorable band positions of the valence band and conduction band of the prepared high-entropy (Ce_0.2_Pr_0.2_Zn_0.2_Nd_0.2_Tb_0.2_)_2_Zr_2_O_7_ zirconate pyrochlore oxide photocatalyst could degrade dyes and reduce toxic Cr(VI), demonstrating its multifunctional applications, and the proposed scheme is displayed in Figure 8.

(1) $$\left(UV\;light\right)hʋ+{\left({Ce}_{0.2}{Pr}_{0.2}{Zn}_{0.2}{Nd}_{0.2}{Tb}_{0.2}\right)}_{2}{Zr}_{2}O_{7}\;photocatalyst\;\to e_{CB}^{-}+h_{VB}^{+}\;$$

(2) eCB−+H2O2→+O•H+OH−

(3) H2O+hVB+→O•H+H+

(4) $$O_{2}+e_{CB}^{-}\;\to \;O_{2}^{•-}$$

(5) O•H/O2•−+MB dye/CR dye→Degradation product

(6) $$HCOOH+h_{VB}^{+}\to CO_{2}+2H^{+}$$

(7) $$HCrO_{4}^{-}+7H^{+}+3e_{CB}^{-}\to {Cr}^{3+}+4H_{2}O$$

### 3.4. Photocatalyst Recyclability and Stability

The recyclability and stability of the prepared photocatalyst were investigated for three repetitive cycles. At the end of the first cycle, the photocatalyst was recovered from the reaction solution by centrifugation (5000 rpm for 10 min). The resultant powder was washed several times with DI and dried at 80 °C overnight in an oven and used as such for the subsequent cycles. The degradation efficiency was calculated using (Equation (8)):

(8) $$Degradation\;\left(\%\right)=\;\left(\frac{C_{0}-C}{C_{0}}\right)*100$$

**Figure 9:**
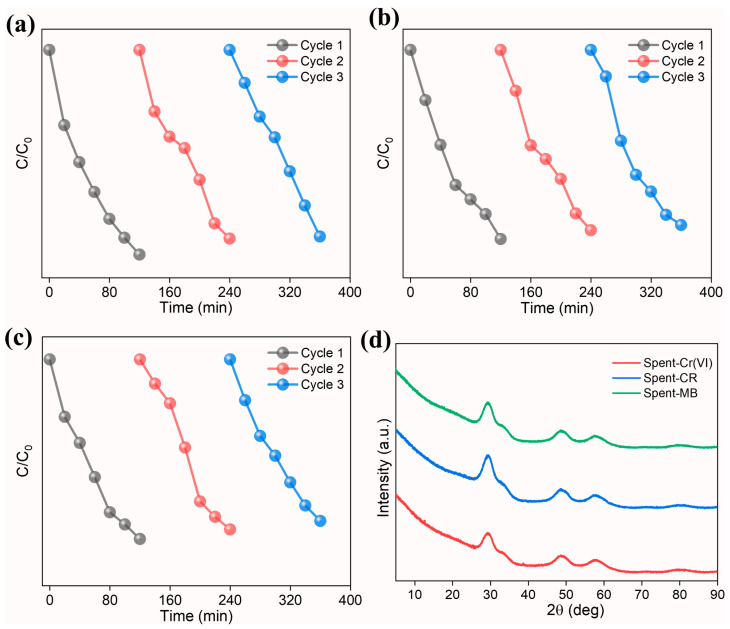
Normalized concentration (C/C_0_) as a function of irradiation time for three consecutive cycles using high-entropy (Ce_0.2_Pr_0.2_Zn_0.2_Nd_0.2_Tb_0.2_)_2_Zr_2_O_7_ pyrochlore zirconate oxide photocatalyst (**a**) Cr(VI), (**b**) Congo red, and (**c**) MB. (**d**) XRD pattern of spent photocatalyst.

The results indicate that the photocatalyst achieved good degradation performance (Figure 9a–c). We observe a marginal decline in the degradation (Table 5) in all three cases, which can be ascribed to the leaching of the photocatalyst during the recovery process. The phase stability of the spent photocatalyst (Figure 9d), characterized using XRD studies, reveals good phase stability without any phase separation. The studies highlight the use of high-entropy (Ce_0.2_Pr_0.2_Zn_0.2_Nd_0.2_Tb_0.2_)_2_Zr_2_O_7_ pyrochlore zirconate oxide as a photocatalyst towards effective degradation of dyes and reduction in Cr(VI).

## 4. Conclusions

In the present work, we prepared a single-phase high-entropy zirconate pyrochlore oxide with composition (Ce_0.2_Pr_0.2_Zn_0.2_Nd_0.2_Tb_0.2_)_2_Zr_2_O_7_ using a simple Pechini method. The prepared high-entropy oxide was characterized to investigate the crystal phase, morphology, elemental composition and distribution, and optical properties. The results indicate that the prepared high-entropy (Ce_0.2_Pr_0.2_Zn_0.2_Nd_0.2_Tb_0.2_)_2_Zr_2_O_7_ zirconate pyrochlore oxide was phase pure, while the bandgap of 2.72 eV was obtained. The prepared high-entropy (Ce_0.2_Pr_0.2_Zn_0.2_Nd_0.2_Tb_0.2_)_2_Zr_2_O_7_ zirconate pyrochlore oxide was used as a photocatalyst to degrade various pollutants such as Cr(VI), Congo red, and methylene blue dye. The photocatalysts performed better in degrading Congo red and methylene blue and reducing Cr(VI) separately. A radical trapping experiment underscores the involvement of hydroxyl radicals, superoxide radicals, and holes in the degradation of dyes and the reduction in Cr(VI). Notably, the photocatalyst could be used effectively three times, and the leaching of the photocatalyst during the recovery step dampened the subsequent degradation activity. However, the recycled photocatalyst was structurally stable without any phase separation, retaining its single phase. The study will serve as a guide for constructing prospective photocatalysts based on the principle of high entropy, and the desired characteristics may be tuned to meet specific application requirements. Beyond that, the prepared high-entropy (Ce_0.2_Pr_0.2_Zn_0.2_Nd_0.2_Tb_0.2_)_2_Zr_2_O_7_ zirconate pyrochlore oxide could be a potential candidate for other functional applications, such as thermal barrier coatings, and requires possible exploration.

## Figures and Tables

**Table 1:** Rietveld refinement results of high-entropy (Ce_0.2_Pr_0.2_Zn_0.2_Nd_0.2_Tb_0.2_)_2_Zr_2_O_7_ zirconate nanoparticles.

Sample	R_p_ (%)	R_wp_ (%)	R_exp_ (%)	χ^2^	Lattice Parameter (Å)
**(Ce_0.2_Pr_0.2_Zn_0.2_Nd_0.2_Tb_0.2_)_2_Zr_2_O_7_**	10.1	9.34	7.93	1.39	10.594

**Table 2:** EDS quantification of metal cations and anions present in (Ce_0.2_Pr_0.2_Zn_0.2_Nd_0.2_Tb_0.2_)_2_Zr_2_O_7_ nanoparticles.

Site	Element (Atomic %)						
	Ce	Pr	Nd	Tb	Zn	Zr	O
**Site 1**	2.48	2.16	2.75	2.22	2.33	12.55	75.52
**Site 2**	3.6	3.19	3.52	2.49	3.11	13.36	70.73
**Site 3**	2.69	2.59	2.99	2.19	2.26	11.9	75.39
**Site 4**	2.68	2.23	2.63	2.05	2.38	11.73	76.29
**Site 5**	2.69	2.75	3.43	2.51	2.62	12.87	73.14
**Mean**	2.82	2.58	3.06	2.29	2.54	12.48	74.21
**S.D.**	0.39	0.37	0.35	0.17	0.30	0.60	2.03

S.D.—Standard deviation.

**Table 3:** Pseudo-first-order reaction kinetics of various dyes and Cr(VI).

Pollutant	Cr(VI)	Congo Red	Methylene Blue
**Rate Constant k (min^−1^)**	0.0506	0.0183	0.0128

**Table 4:** Comparison of photocatalytic degradation of dyes and reduction in Cr(VI) with reported photocatalysts.

Photocatalyst	Dye	Irradiation Source	Dye/Cr(VI) Concentration	Photocatalyst Concentration	Irradiation Duration (min)	k min^−1^	Reference
ZnO-QDs/In_2_O_3_ nanofibers	Cr(VI)	Visible light	50 µM/L	500 mg/L	120	0.018	[6]
Zn QDs/ZnWO_4_		400 W metal halide lamp	20 mg/L	100 mg/L	40	0.0599	[8]
SW-2		500 W Xe lamp	50 mg/L	1 g/L	40	0.075	[35]
Bi_2_S_3_/Bi		300 W xenon lamp	20 mg/L	500 mg/L	30	0.0246	[4]
Ti–60Nb		UVC germicidal lamp (TUV 54 W, Philips; 254 nm)	20 ppm	-	120	0.0167	[2]
4 % Zn-CoFe_2_O_4_		Visible light	20 mg/L	333 mg/L	135	0.025	[36]
(Ce_0.2_Pr_0.2_Zn_0.2_Nd_0.2_Tb_0.2_)_2_Zr_2_O_7_		UV-LED light	50 ppm	500 mg/L	120	0.0506	This work
TiO_2_-g-C_3_N_4_-10	CR	Sunlight	50 mg/L	50 mg/L	160	0.0259	[37]
ZnO/CNT/GO		LED light	10 ppm	600 mg/L	60	0.0253	[38]
0.5-BiOI/BOC		Xenon lamp	100 mg/L	30 mg/L	60	0.0207	[39]
BiOI/GO		100 W tungsten iodide lamp	20 mg/L	200 mg/L	120	0.02151	[10]
C2B2LF		Sunlight	10 mg/L	1000 mg/L	120	0.0093	[40]
GdCo_0.80_Mn_0.20_O_3_		200 W xenon lamp	10 ppm	600 mg/L	120	0.0079	[41]
(Ce_0.2_Pr_0.2_Zn_0.2_Nd_0.2_Tb_0.2_)_2_Zr_2_O_7_		UV-LED light	50 ppm	500 mg/L	120	0.0183	This work
BV3	MB	Visible light	1 mg/L	80 mg/L	120	0.00592	[42]
ZG nanohybrids		UV–visible light	1 ppm	-	130	0.048	[43]
JC-Cu_0.05_Zn_0.95_O		250 W mercury vapor lamp	10 mg/L	1000 mg/L	120	0.01831	[44]
Mo–Bi_2_WO_6_/WO_3_/Biochar		500 W halogen lamp	6.4 mg/L	600 mg/L	30	0.02816	[5]
1 wt% Ca-doped TiO_2_		UV-visible irradiation	20 ppm	800 mg/L	180	0.0087	[11]
BVO/CWB-11		300 W xenon lamp	20 mg/L	1000 mg/L	120	0.02732	[45]
(Ce_0.2_Pr_0.2_Zn_0.2_Nd_0.2_Tb_0.2_)_2_Zr_2_O_7_		UV-LED light	50 ppm	500 mg/L	120	0.0128	This work

**Table 5:** Recyclability studies using the prepared (Ce_0.2_Pr_0.2_Zn_0.2_Nd_0.2_Tb_0.2_)_2_Zr_2_O_7_ oxide towards the degradation of MB, CR, and reduction in Cr(VI).

Pollutant	Degradation/Reduction (%)		
	Cr(VI)	Congo Red	Methylene Blue
**Cycle 1**	98.8	91.3	79.3
**Cycle 2**	91.1	87.1	75.1
**Cycle 3**	90.1	84.6	71.4

## Data Availability

The original contributions presented in this study are included in the article. Further inquiries can be directed to the corresponding author.

## Abbreviations

The following abbreviations are used in this manuscript:

AOP	Advanced oxidation process
BOD	Biochemical oxygen demand
CA	Citric acid
CR	Congo red
DI	Deionized water
EDS	Energy-Dispersive X-ray Spectroscopy
EDTA-2Na	Ethylenediaminetetraacetic acid disodium salt
EG	Ethylene glycol
FESEM	Field emission scanning electron microscopy
HEMs	High-entropy materials
ICDD	International Centre for Diffraction Data
IPA	Isopropyl alcohol
K-M	Kubelka-Munk
MB	Methylene blue
NHE	Normal Hydrogen Electrode
p-BQ	p-benzoquinone
ROS	Reactive oxygen species
S.D.	Standard Deviation
UPS	Ultraviolet photoelectron spectroscopy
W-H	Williamson-Hall
XPS	X-ray photoelectron spectroscopy
XRD	X-ray diffraction
